# Effect of Functionalized Graphene Nanoplatelets on the Delamination-Buckling and Delamination Propagation Resistance of 3D Fiber-Metal Laminates Under Different Loading Rates

**DOI:** 10.3390/nano9101482

**Published:** 2019-10-18

**Authors:** Davide De Cicco, Farid Taheri

**Affiliations:** Advanced Composites and Mechanics Laboratory, Department of Mechanical Engineering, Dalhousie University, 1360 Barrington Street, P.O. Box 15 000, Halifax, NS B3H 4R2, Canada; davide.decicco@dal.ca

**Keywords:** 3D fiber-metal laminates, graphene nanoplatelets, impact buckling, delamination buckling, delamination propagation, temperature effect

## Abstract

This paper presents an investigation into the effect of graphene nanoplatelets (GNPs) as a means of improving the impact buckling performance and delamination propagation resistance of a recently developed 3D fiber-metal laminate (3D-FML). One of the highlights of the investigation is the examination of the performance of the GNP-reinforced resin at a sub-freezing temperature (−50 °C). 3D-FML beam specimens were subjected to axial impact of various intensities at room-temperature, while they were subjected to quasi-static axial compression load at the sub-freezing temperature. Moreover, the influence of two different surface preparation methods on the performance of the metallic/FRP interfaces of the hybrid system was also investigated in this study. Although the inclusion of the GNPs in the resin resulted in some gain in the buckling capacity of the 3D-FML, nevertheless, the results revealed that the lack of adequate chemical bond between the GNP-reinforced resin and the magnesium skins of the hybrid material system significantly limited the potential influence of the GNPs. Therefore, a cost-effective and practical alternative is presented that results in a significant improvement in the interfacial capacity.

## 1. Introduction

**Figure 1:**
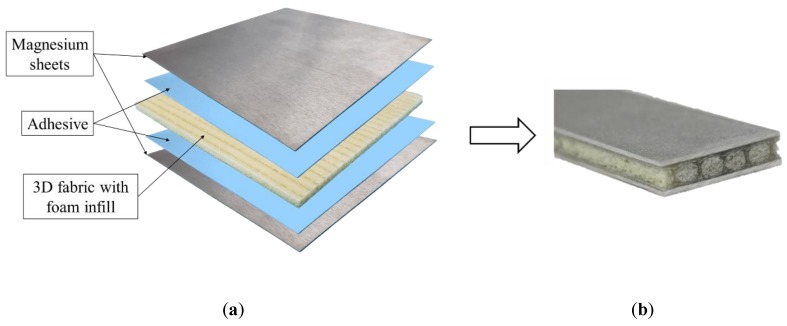
The 3D-FML composite: (**a**) exploded view, showing the various components, and (**b**) the final product.

The automobile industry, like many other industries, is facing challenges in complying with the recent and continually increasing strict environmental regulations and safety requirements. Therefore, the development of cost-effective performant materials, ranging from light-weight metallic alloys to various composites, are being increasingly explored to improve vehicles’ fuel consumption and passenger safety. In response, new metallic alloys’ compositions are becoming increasingly complex in order to optimize their performance (cf. [1,2]). However, one of the proven strategies for developing light-weight hybrid materials has been shown to be attained by the marriage of lightweight metal alloys and advanced fiber-reinforced polymers (FRPs), referred to as fiber-metal laminates (FMLs). Following this path, our research group recently developed a new class of FML, consisting of a truly three-dimensional hollow-core fiberglass fabric, with its core filled with a light-weight foam, sandwiched between thin sheets of a lightweight magnesium alloy. This hybrid composite system, shown in Figure 1, is referred to as 3D-FML. Due to the impressive specific strength, stiffness and impact absorption properties of this hybrid system, 3D-FMLs are considered as economical and effective light-weight material systems, suitable for the fabrication of transport vehicles and aircraft shell structures [3,4]. However, as highlighted in some of the authors’ previous works [5,6,7], the outstanding performance of this class of FML is somewhat compromised when the system becomes subject to in-plane loading. This is mainly due to the relatively low strength of the magnesium/FRP interface segment of this FML. In other words, in general, the interface strength between the metallic and FRP constituents is the Achilles’ heel of essentially all classes of FMLs. Therefore, various methods and strategies have been explored to increase the interfacial bond strength; these approaches can be essentially divided into the following categories: (i) abrasion [8,9,10], (ii) chemical etching (which includes plasma surface activation) [11,12,13,14], (iii) use of nanoparticles to reinforce the interface resin, and (iv) a combination of the aforementioned methods.

Abrasion enhances the mechanical bond (interlocking) between the substrate and the resin, while chemical etching improves the chemical bond between the two interfacing materials. The use of nanoparticles (NPs), however, strengthen the resin used for bonding the two substrates and bridges the potential micro-cracks and their growth, as well as enhancing the interlocking between the substrates’ micro-cavities and the adhesive. It is worth noting that NPs themselves cannot enhance the chemical bond between the adhesive and substrate. Therefore, in the absence of an effective chemical bond, the effectiveness of mechanical interlocking becomes significantly compromised [15].

In this paper, the influence of incorporation of graphene nanoplatelets (GNPs) on enhancing the interfacial bond strength is investigated. It should be noted that other effective types of nanoparticles are also available for the purpose (see for instance the use of nanosilica [16,17,18], nanoclay [19,20,21], polymers themselves [22,23], and, for specific medical applications, nanogold and nanosilver, [24,25,26]). Several studies have shown that the modulus of elasticity, tensile strength, and fracture toughness [27,28,29,30,31,32,33,34], as well as fatigue resistance [35,36] and vibration damping capacity [37,38,39,40] of polymers could be positively enhanced by the incorporation of appropriate NPs. NPs have also been shown to improve the bond strength of adhesively bonded joints, especially in lap-strap joints [27,41,42].

Improvements in impact resilience gained by the use of NPs have also been demonstrated. Haro et al. [43] and Áliva et al. [44] performed ballistic impact tests on a Kevlar/aluminum FML and fiberglass/epoxy composite, respectively. They observed that alumina, silica and nanoclay NP-reinforcements, respectively, led to increasing ballistic protection; however, the inclusion of the NPs caused additional delamination extension. Haq et al. [45] performed low-velocity impact tests on sandwich composites. They reinforced the fiberglass/epoxy facial laminates by coating them with graphene. The authors concluded that an optimal spatial distribution of NPs could be done to optimize the response of components subjected to impact. Some researchers have also shown that GNPs were more effective than carbon nanotubes (CNTs) in delamination mitigation and in arresting crack propagation [46,47]. For instance, Rafiee et al. [48] demonstrated that while the incorporation of CNTs in epoxy led to a 20% increase in the fracture toughness, a 53% increase was obtained when GNPs was used as the reinforcement. In addition, Ahmadi-Moghadam et al. [49,50] demonstrated that further improvements in fracture toughness (in all three modes) could be attained through functionalization of GNPs. Functionalization is a chemical process aimed at improving the interfacial bond between NPs and resins. The authors showed that amongst the four different functionalization schemes they tried, the best results were obtained when NH_2_ was used to functionalize the GNPs.

There are, however, studies that report the incorporation of NPs did not lead to beneficial outcomes. For instance, Wichman et al. [51] observed some increase in the mechanical properties of their CNT-reinforced fiberglass/epoxy, however, no improvement in the delamination resistance was attained. Using the same type of NPs, Siegfried at al. [52] reported an increase in impact performance of carbon fiber/epoxy composites subjected to low-velocity impact, but at the expense of increased delamination. The authors attributed the loss of the interlaminar strength to an increase in the matrix brittleness due to the incorporation of NPs. Bortz et al. [53] reported an increase in the stress intensity factor of 63% when GNPs were incorporated into the resin, thus, showing how the composite was more prone to cracking.

Since this paper focuses on the compressive axial behaviour of 3D-FML, it is worth mentioning some of the notable works related to the study of buckling in composites, more specifically, related to composites that have a delamination. The presence of a delamination in composites has been shown to negatively affect their response, especially when the composite is subjected to an in-plane compressive loading [54,55,56,57,58]. Delamination can be initiated in FRP due to even a low-energy impact (i.e., caused by the impact of a falling tool during fabrication) and/or other manufacturing induced flaws. Esfahani et al. [59] carried out a numerical study and showed that the presence of a delamination had a negative impact on the buckling capacity of their specimens, especially when the delamination was close to the specimen’s outer laminae. Kim and Hong [60] reported that the length and position of the delamination were two parameters that had a large influence on the buckling mode and post-buckling behaviour of laminated composites. It should, however, be noted that there exists a threshold under which the delamination length would not affect the buckling strength of composites. Asaee et al. [61] demonstrated the efficacity of using GNPs in improving the in-plane static compression response of short 3D-FML beams. By adding the GNPs to the bonding interface between the magnesium and core part of their hybrid material system, they observed up to 25% increase in the specimens’ load-bearing capacity.

When considering transport vehicles, in addition to the conventional mechanical loads (including impact loads), they also become subjected to severe environmental conditions, including extreme temperatures and humidity. In many areas in the world, temperatures as low as −50 °C are commonly reached and maintained during the winter period. Therefore, it is important to assure the durability of materials used in fabricating transport vehicle panels, especially in circumstances when the vehicle becomes subjected to an impact within the harsh environment. Several studies have investigated the effect of temperature on the performance of composite materials. For example, Taraghi et al. [62] subjected Kevlar/epoxy composite to low-velocity impacts at room and sub-freezing (−40 °C) temperatures and observed 35% and 34% reduction in damage density, respectively, as a result of the inclusion of CNTs to their epoxy resin. Shen at al. [63] showed that the inclusion of graphene oxide particles improved the interlaminar shear strength of fiberglass/epoxy composites by 32% at a cryogenic temperature of 77 K (−196 °C). A few authors have also considered the influence of thermal cycles on materials [64,65]. For instance, Khalili et al. [66] investigated the influence of moisture and the subsequent thermal cycles on the strength of hybrid bonded/bolted joints mating FML substrates. The specimens were initially soaked in seawater for 30 days and were subsequently subjected to 10 thermal cycles (between −40 °C to −100 °C). They observed a 35% reduction in the strength of their immersed specimens. However, the cooling cycles recuperated 50% of the lost strength in their joints. This gain in the strength was believed to have occurred as a result of the relaxation of the residual stresses developed in the immersed specimens.

In this paper, the in-plane compression and impact-buckling responses of 3D-FML whose metal/FRP interfaces are reinforced with NH_2_-functionalized GNPs are investigated. In particular, the influence of the existence of a delamination in the FML is also considered. The responses of reinforced specimens are compared against the baseline specimens (i.e., with non-reinforced specimens). Moreover, the influence of sub-freezing temperature (−50 °C) on the performance of the non-reinforced and GNP-reinforced interfaces subjected to quasi-static compression loading is also investigated.

## 2. Materials and Methods

### 2.1. Materials

The 3D fiberglass fabric and fiberglass veil were acquired from China Beihai Fiberglass Co. Ltd. (Jiujiang City, Jiangxi, China). A Huntsman produced two-part hot-cure epoxy resin (bisphenol-A-based Araldite LY1564 resin and its Aradur 2954 hardener) was acquired from the producer (Huntsman Corporation, West Point, GA, USA), while the cold-cure epoxy resin (105 resin with 206 hardener) used to mate the magnesium and FRP constituents (i.e., the interface region herein) was produced by West System (Bay City, MI, USA). An 8-lb/ft^3^, high-density polyurethane foam was obtained from US Composites (West Palm Beach, FL, USA). The NH_2_ functionalized graphene nanoplatelets (hereafter referred to as GNPs for the sake of brevity), having an in-plane dimension of 1–2 μm and thickness of 4 nm, were purchased from CheapTubes Inc. (Cambridgeport, VT, USA). The lightweight AZ31B-H24 magnesium alloy sheets (or skins) were acquired from MetalMart (Commerce, CA, USA). Finally, liquid nitrogen was obtained locally.

### 2.2. Specimen’s Fabrication

**Figure 2:**
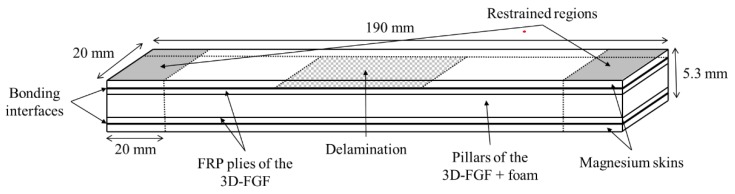
Schematic illustration of the 3D-FML specimen hosting a delamination and its overall dimensions (drawing not to scale).

All the beam-like specimens, schematized in Figure 2, with dimensions of 190 mm × 20 mm × 5.3 mm, were extracted from larger 3D-FML plates, using a water-cooled circular saw equipped with a diamond-coated blade. The sequence of procedures used to fabricate the plates is as follows. The two parts of the hot-cure epoxy resin were mixed at 100 rpm for 10 min. using a mixer, then degassed in a vacuum chamber for a minimum of 30 min. Then, the mix was applied homogeneously onto the 4 mm thick 3D fiberglass fabric (3D-FGF) using a brush. The resin-immersed fabric was cured at 60 °C for two hours and subsequently at 120 °C for 8 h, after which the fabric took its three-dimensional configuration with cavities in its core (see Figure 1). The cavities were then filled with the foam to provide support to the thin pillars connecting the two main biaxial E-glass constituents of the fabric, thereby increasing the overall specimen’s stiffness and strength. The foam-filling process was done by drawing the foam into the cavities at its liquid stage under a negative pressure using an in-house designed jig, which guarantees a homogeneous repartition of the foam inside the cavities. The combination of the 3D fabric-epoxy and foam will be referred to as “core” hereafter. 

The hybrid sandwich composite system was completed by bonding the magnesium plates (skins) to the core. Two bonding methods were used, thus leading to two different categories of specimens. In both methods, first, the magnesium skins’ bonding surfaces were sandblasted with coarse 20–30 grit crushed glass abrasive in order to facilitate good mechanical bonding. The two bonding methods are as follows:

(i) in the first method (referred to as SB, hereafter), the hot-cure resin was directly applied onto the substrates (skins and core), and then the resulting sandwich was vacuum bagged and cured for two hours at 60 °C and eight hours at 120 °C.
(ii) in the second method (referred to as SBC, hereafter), the magnesium bonding surfaces were pre-coated with a thin layer of cold-cure resin, cured for 24 h under vacuum. In a second step, another layer of cold-cure resin was applied to both adherends and they were sealed under vacuum and let cure at room temperature for 24 h. This second method was developed by the authors and the resulting gain in the interface bond strength under different loading conditions, including axial impact loading, was reported in [67].

For the specimens hosting a delamination, a thin sheet of Teflon was inserted between the magnesium and the core during the bonding process. The resulting delamination had a length of 30%, 50% or 70% of the specimens’ gage length, and were placed at mid-length, on one of the interfaces only (see Figure 2).

In some specimens, GNPs were incorporated into the resin that was used to adhere the skins to the core. This was done according to the detailed procedure described in [42]. In brief, the various weight percentages (wt%) of the functionalized-GNPs nanoparticles were accurately measured using a scale having a precision of 0.5 mg. The GNPs were mixed with the cold-cure resin (part 105) using a variable speed mixer for 15 min. with an initial speed of 400 rpm, gradually increasing to 2000 rpm. Then, the mixture was further homogenized by passing it seven times through a three-roll calendering machine to break the agglomerations and facilitate uniform dispersion of the particles. Finally, the hardener (part 206) was incorporated, and the whole mixture was mixed at 400 rpm for four minutes and subsequently degassed for five minutes. The short mixing and degassing times prevent the resin from partially curing before it is applied onto all the adherends’ surfaces. After degassing, the resin was used as an adhesive in the same manner as described earlier.

### 2.3. Testing Apparatus, Procedures and Data Acquisition

#### 2.3.1. Case Studies I

**Figure 3:**
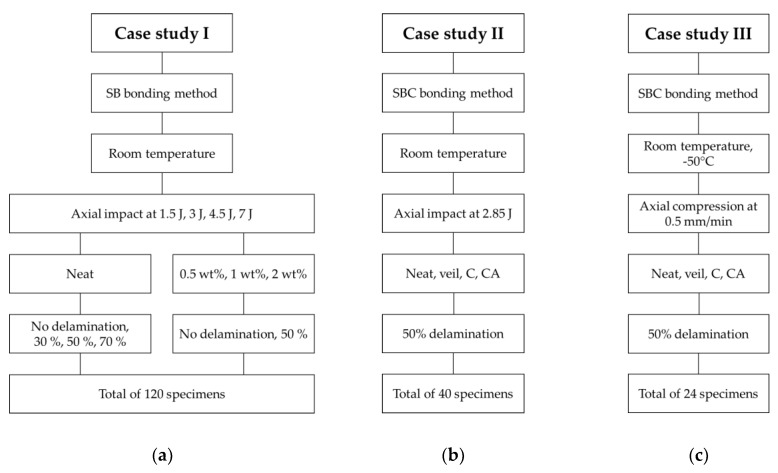
Summary of the case studies and their parameters: (**a**) I, (**b**) II, and (**c**) III.

The experimental investigation of this study was organized within three distinct case studies (I, II, and III), as summarized in Figure 3.

In the first study, the integrity of the SB bonding method and the effect of GNP inclusion on the performance of the interface bond was studied. Specimens used in this category were fabricated with four different GNP contents (i.e., no GNPs (referred to as “neat” and identified by “N”), 0.5 wt%, 1 wt% and 2 wt% contents). Each specimen category was subjected to four impact energies (1.5 J, 3 J, 4.5 J, and 7 J). The impact energies were chosen according to an experimental investigation conducted earlier by the authors [6] and are aimed to cause (i) elastic buckling; (ii) initiation of a permanent deformation; (iii) propagation of the delamination and (iv) complete failure of the specimens, respectively.

Four initial delamination scenarios were considered for the neat specimen group; they were: intact (i.e., with no initial delamination), identified as ND; and those with three different initial delamination lengths equal to 30%, 50% and 70% of the specimens’ gage length. For the specimens that contained GNPs, only the intact specimens and the specimens with initial delamination length of 50% were considered. Detailed justification of the selection criteria is provided in the next sections.

It should be noted that the effect of the inclusion of GNP on the interface bond strength (i.e., case study I) was performed previously. However, as will be explained in detail in the subsequent section, the benefits that could be gained by the inclusion of GNPs in the resin were rendered inconclusive. Therefore, the new bonding procedure described in the previous section was used to form the case studies II and III.

#### 2.3.2. Case Study II

For the second case study, one delamination length (i.e., 50% of the gage length), one impact energy (2.85 J), and one GNP content (0.5 wt%) were considered. In this way, more statistical number of specimens could be considered per testing category. The 2.8 J impact energy was selected on the basis that it would cause partial buckling of the specimens and propagating the initial delamination while preventing the complete failure of the specimens. Note that the complete delamination of the skin would defeat one of the objectives of the study (i.e., the examination of GNP’s effect on the delamination growth). Furthermore, the inclusion of GNPs in the resins were done in two ways:

(i) included only in the resin used to coat the magnesium skins was reinforced with the GNPs (these specimens are identified as “C” specimens);
(ii) included in the resin used to coat the skins and in the resin used for bonding the skins to FRP were both reinforced with the GNPs (specimens of this category are identified by “CA”).

Aside from the GNPs, the effect of inserting a thin fiberglass veil between the magnesium and the core with the aim of improving the interface bond mechanism was also investigated (specimens in this category are identified as “V” specimens). Finally, the baseline specimens, which were fabricated with the neat resin (i.e., with no GNP or veil reinforcements) are identified as “N” specimens.

#### 2.3.3. Impact Testing Apparatus

**Figure 4:**
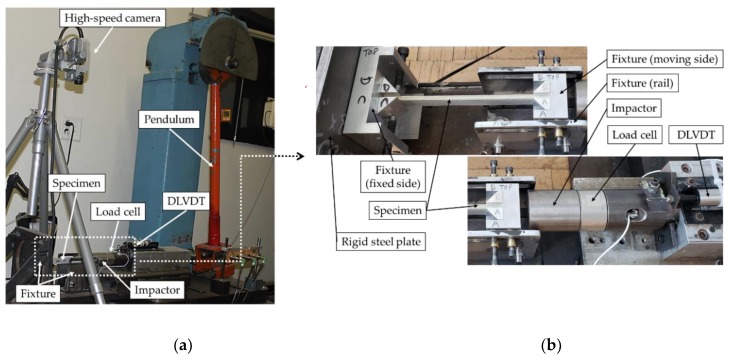
(**a**) Experimental setup for impact testing and (**b**) close-up view of the impactor and the specimen supported by the fixture.

The impact testing apparatus used to test the specimens of case studies I and II is shown in Figure 4. The setup consisted of a modified Charpy impact testing machine equipped with an in-house designed fixture to support the specimen such that a given specimen would be subjected to a purely axial impact. Each specimen was clamped in the fixture over 20 mm length at each end in such a way that only the axial displacement at the impacted end was permitted, therefore, imposing a fixed-fixed boundary condition. The various impact energies were obtained through trial and error, by changing the pendulum angle and using an image-processing algorithm written in MATLAB, to extract the position and time information of the impactor. The impact load and axial-shortening history data were captured using a dynamic load cell and a dynamic linear variable displacement transducer (DLVDT), respectively, both operated at a sampling rate of 50 kHz. The signals were transferred to a PC via a National Instruments data acquisition system device, synchronized using the Signal Express software. A Photron Fastcam PCI high-speed camera was used to record the impactor movement and specimens’ deformation at a rate of 2000 fps for the first case study, while a Kronotek Chronos high-speed camera was used for the case study II tests, at a rate of 4498 fps. Note that the latter camera was not available to the authors at the time the first case study was conducted (hence, the use of two different cameras).

#### 2.3.4. Case Study III

The same parameters that were used in case study II were used in case study III, but the tests were conducted under quasi-static loading (cf. Figure 3). This is because the sub-freezing temperature had to be conducted in an Instron thermal chamber that could not accommodate the impact test setup. 

**Figure 5:**
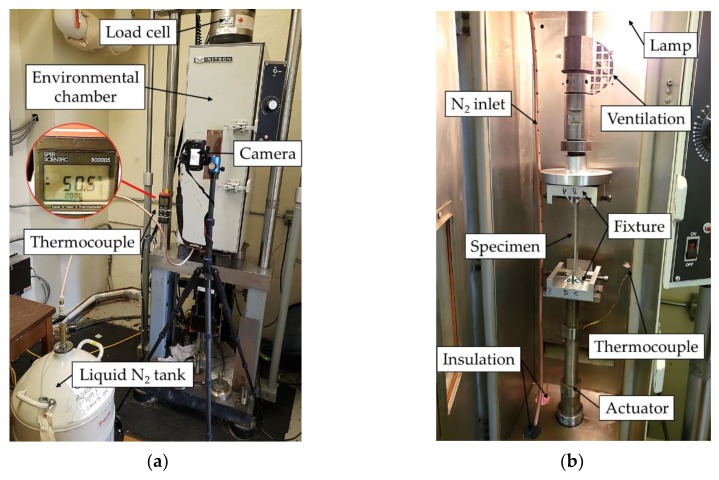
(**a**) Overall view of the static buckling test setup and (**b**) inside view of the thermal chamber.

The chamber was used in conjunction with an MTS servo-hydraulic testing machine, equipped with a 250 kN load cell. This test setup is illustrated in Figure 5. The compression actuation speed was set to 0.5 mm/min. The air inside the chamber was cooled down to −50 °C using liquid nitrogen, and the temperature was monitored using a thermocouple. Finally, the load and displacement data were retrieved directly from the MTS machine using the MTS793 software that was used to control it, while the delamination-buckling event was captured on video at a rate of 30 fps using a Rebel SL2 camera (Canon, Tokyo, Japan).

### 2.4. Data Processing

**Figure 6:**
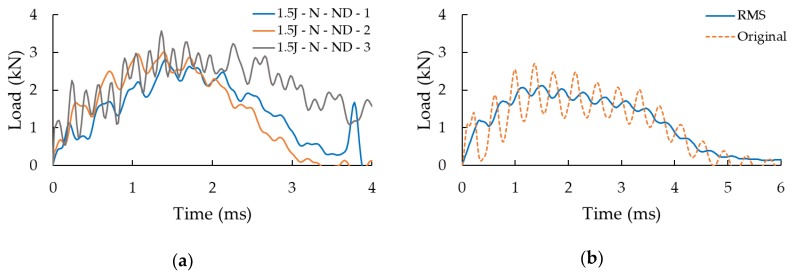
(**a**) The raw load history signals of neat specimens (i.e., without al delamination) impacted at 1.5 J, (**b**) A typical filtered signal and its RMS.

A LabVIEW algorithm was developed to facilitate the post-processing of the impact test data in a consistent and efficient manner. The only required operation of the user was the identification of the exact initial time of the impact event. The output of the LabVIEW code was a set of data points corresponding to three signals. First, the captured displacement-time signal was filtered to remove high-frequency noise and the 60 Hz noise originated by the power supply, followed by filtering of the force-time signal. An example of such signals is illustrated in Figure 6a. Moreover, since the inherent signal fluctuation makes it difficult to objectively compare the signals obtained from testing various specimens, therefore, the RMS (acronym of the root-mean-square) of the signals was obtained, as illustrated in Figure 6b. The RMS data was established by evaluating the average of the load-history signal, computed using the RMS amplitude of the signal. This quantity is directly proportional to the signal’s power and peak amplitude. Therefore, the information conveyed through the RMS signal is equivalent to the one from the filtered signal from which they are extracted. The application of this signal-processing procedure would not be necessary when analyzing the quasi-static test results, since there would be no such inherent fluctuation in the signals in such tests.

To measure the delamination growth, the initial delamination was precisely measured using a digital microscope and its extremities were marked using a permanent marker. Then, clearly visible tick marks, spaced at 5 mm intervals, were inscribed along the specimens’ side. The delamination growth in each specimen was then measured by comparing the images (extracted from the videos) of the specimen at its initial and deformed states.

## 3. Results and Discussion

In this section, the results of the three case studies are reported and discussed. For the sake of clarity and brevity, only the response of a typical specimen per group of specimens will be illustrated, with the proviso that the exhibited curves are close representatives of the response of all specimens tested within each specimen group. The level of consistency in typical date is illustrated in Figure 6a.

### 3.1. Case Study I

**Figure 7:**
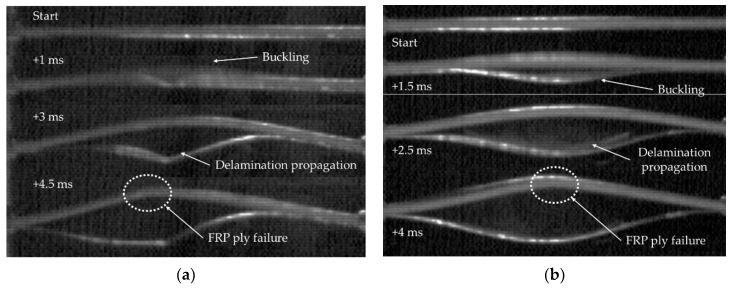
Illustration of the behaviour of the 3D-FML sandwich under axial impact, for the study case I neat specimens. (**a**) No initial delamination, 7 J and (**b**) 50% initial delamination, 4.5 J.

Typical qualitative responses of an intact (neat) and a specimen having a delamination are shown in Figure 7. In general, the specimens remained straight during the first instance upon the application of the impact load, regardless of the considered impact energies. The intact specimens subsequently experienced global buckling. The specimens that were subjected to 1.5 J impact energy endured the energy by elastic deformation and fully recovered their original status after the event. Those undergoing 3 J impact energy, also underwent global buckling, however, ending up with a permanent deformation since their magnesium skins endured some degree of plastic deformation. The behaviours of the specimens undergoing 4.5 J impact energy was similar to those that were subjected to 3 J impact energy, with the difference that one of the skins partially delaminates in this category. Finally, for 7 J impact energy case, the specimens’ skin, on the side that underwent compression during the buckling event delaminated, and the FRP plies of the 3D-FGF on the compression side crushed, leading to the complete failure of the specimens.

The specimens having a delamination experienced a global buckling mode; however, during the buckling, the delaminated portion of the skin also experienced local buckling. The delamination then grew to a certain extent depending on the applied impact energy. The propagation of delamination was observed to be marginal in the specimens that were subjected to the lowest impact energy. However, the delamination propagated along the entire span of the specimens that were subjected to 3 J impact, but their core remained undamaged. Finally, the specimens that experienced 4.5 J energy failed completely (i.e., in addition to complete separation of their skins, their FRP plies also failed in compression).

**Figure 8:**
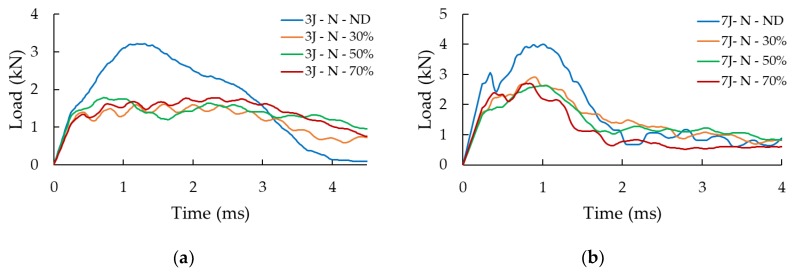
Effect of initial delamination for (**a**) 3 J and (**b**) 7 J cases.

The influence of the presence of a delamination is presented quantitatively in Figure 8 through the load-history graphs of the impact tests performed at 3 J and 7 J on the neat specimens (i.e., specimens without GNPs added to their interfaces). Note that the results of the tests conducted at other two energy levels were omitted for the sake of conciseness since they followed the same pattern. The graphs show a clear reduction of the buckling capacity (corresponding to the maximum load on the curves) for the specimens hosting a delamination compared to the intact specimens. More specifically, reductions in buckling capacity of 26%, 36%, 38%, and 24%, respectively, are observed for specimens experiencing the impact energies of 1.5 J, 3 J, 4.5 J and 7 J. As evident in the curves illustrated in Figure 8, the length of the delamination does not seem to affect the response in a significant manner; in other words, the variation in the impact response is negligible in all three delamination lengths. Consequently, the 30% and 70% delamination cases were not considered for the remaining portion of the study.

**Figure 9:**
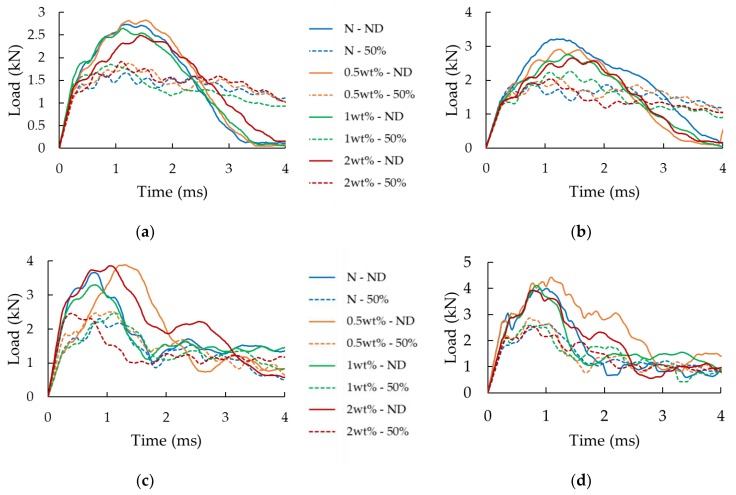
Effect of nanoparticles for specimens with and without delamination, and with and without 0.5 wt% GNP content subjected to the different impact energies: (**a**) 1.5 J, (**b**) 3 J, (**c**) 4.5 J, and (**d**) 7 J.

Figure 9 illustrates the load-history graphs for specimens that were subjected to the four impact energies. As could be expected, a higher impact energy led to a higher measured maximum load-bearing capacity. Overall, the results are more consistent for the lowest and highest energies than for the two medium energies. As discussed in [6], this is attributed to the fact that 3 J and 4.5 J energies hover around the energy that corresponds to the damage threshold. Therefore, the sensitivity to the reaction of a given specimen at the onset of buckling, which is naturally volatile, is further amplified. It can also be seen that the specimens tested at the two higher impact energies appear to exhibit a residual load-bearing capacity. This response is not observed when considering the specimens of the other cases because, in those cases, the load drops to zero when the impactor detaches from the specimen (bounces back). In the 4.5 J impact event, the impactor speed halts to zero but without bouncing, indicating that the impact energy is absorbed fully by the specimens, while under 7 J energy, the specimens are completely crushed by the impact. This shows that once the skins are fully delaminated, the strength of the core is fully compromised.

**Figure 10:**
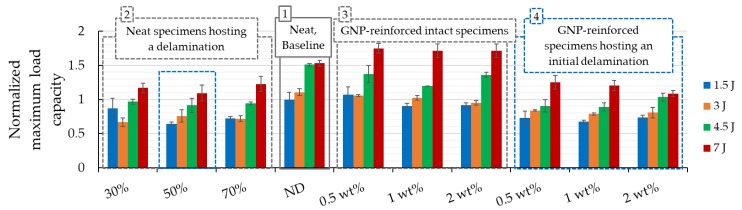
Average normalized buckling capacity for all specimen sets of case study I. Note that the blue boxes identify the comparable specimen categories.

The effects of the GNPs inclusion can also be observed from the results reported in Figure 9, with a more concise comparison illustrated in Figure 10. Note that all the results shown in Figure 10 have been normalized with respect to the performance of the intact neat specimens, which are referred to as the “baseline” specimens hereafter. The standard deviations are also reported in the chart to better quantify the variation in the results.

To provide the reader with a more comprehensive sense of variation is the results, which are somewhat voluminous due to the large number of parameters that were considered, the results are summarized in terms of buckling capacity and reported in Figure 10. In this figure, the results are categorized in numbered boxes for easier comparison. Please note that the capacity is normalized with respect to the baseline specimens (i.e., the specimens without GNPs and without initial delamination, cf. box 1). Box 2 illustrates the results for the specimens that hosted a delamination. As can be seen, there is no distinct difference in the specimens’ response as a function of the initial delamination length. This is attributed to the low bonding strength between the magnesium skins and the composite core, as discussed in a previous study [5].

Within the GNP weight contents considered (cf. box 3), the intact specimens with 0.5 wt% of GNP content show the best overall improved performance under all tried energies, followed by those containing 1 wt% and 2 wt% GNP, respectively. More precisely, we can see that the highest gain (i.e., 12.5% increase in load-bearing capacity) is seen in the specimen that was reinforced with 0.5 wt% GNP, tested under 7 J impact energy. Next in the ranking are the specimens that were reinforced by 1 wt% and 2 wt% GNP contents, exhibiting 10.5% gain in load-bearing capacity. A similar conclusion can be drawn for the specimens that were subjected to 1.5 J impact, but the gains are observed only for the specimen that had 0.5 wt% GNP content. In fact, the nanoparticles seem to have induced a negative effect on the specimens that were subjected to the 3 J and 4.5 J cases, since the specimens’ load-bearing capacity was reduced.

When the influence of initial delamination is considered (cf. box 4), the best results are still shown by the specimens that were reinforced with 0.5 wt% of GNPs, followed by those with 1 wt% GNP content. The specimens containing 2 wt% GNP did not exhibit any gain in their strength. Note that for the case of 3 J, the specimens with 0.5 wt% GNP exhibited good performance, notwithstanding the fact that the outcome is marginally different when compared to the outcomes associated with specimens containing 1 wt% GNP. Similar to the results observed in the case of the intact specimens, GNP inclusion resulted in a detrimental effect when the specimens were subjected to 4.5 J impact energy; however, improvement in performance are also observed in the cases when the applied impact energies were 1.5 J and 3 J. Note that the specimens containing 2 wt% GNP content that were subjected to the highest energy performed the least favourably.

Based on the results, it can be concluded that the addition of GNPs can have beneficial effects on the impact load-bearing capacity of the 3D-FMLs so long as the system has no initial delamination. However, once a delamination is introduced, the lack of an adequate bonding mechanism between the magnesium skin and the resin does not allow the GNPs to play their supportive role in preventing crack initiation and arrest. In the presence of a delamination, the lack of chemical synergy between the magnesium alloy and epoxy resin leads to the catastrophic failure of the interface in the presence of a large magnitude of fracture energy developed by increased loading.

**Figure 11:**
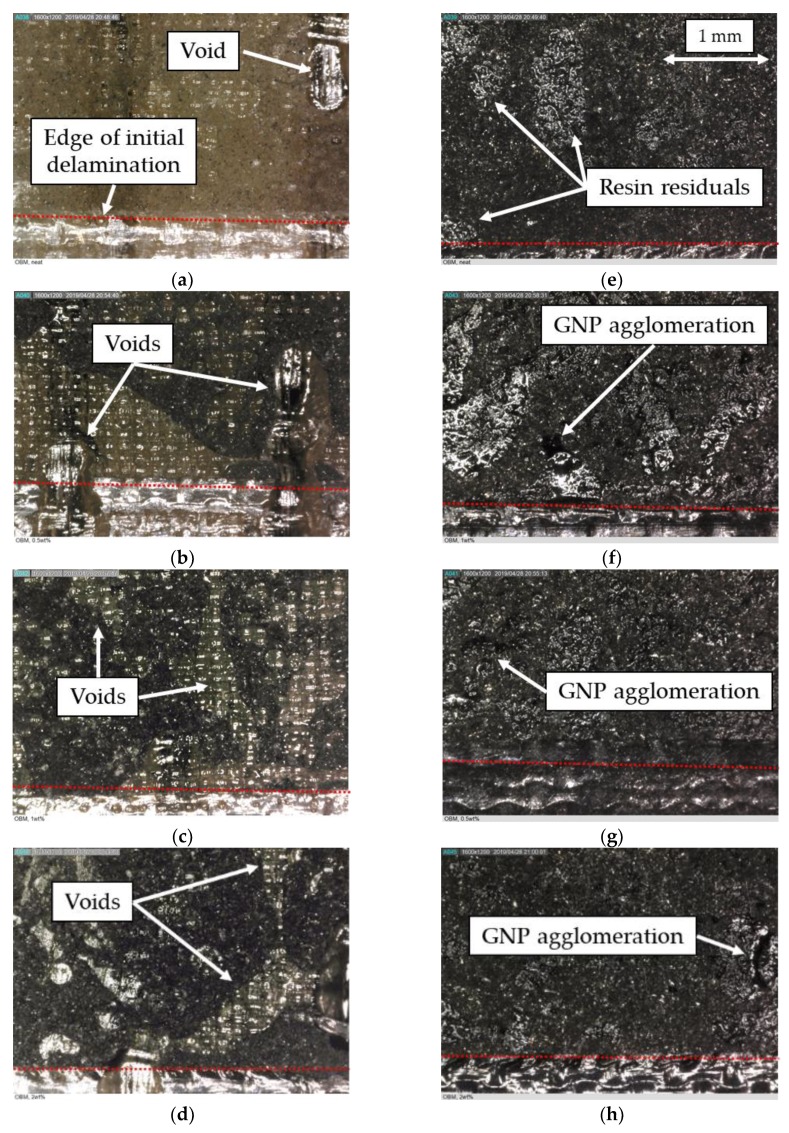
Magnified views of the adherends’ fracture surfaces for specimens of case study I. Images (**a**) to (**d**) show the adhesive interfacial surfaces, while images (**e**) to (**h**) show the magnesium interfacial surfaces. From left to right: neat specimens and specimens with 0.5 wt%, 1 wt%, and 2 wt% GNP contents.

In an attempt to better understand the reason for unanticipated effectiveness of the NPs in suppressing the interface delamination growth of the specimens, the interface bonding surfaces were examined by the use of a digital microscope. The morphology of the surfaces of the specimens within this case study, valid for all the impact energies resulting in delamination, are shown in the micrographs illustrated in Figure 11. One can see that the dispersion of the nanoparticles is homogeneous on the surfaces of the specimens. Note that the darker pixels represent GNPs’ distribution and the lighter colour regions seen at the lower portion of each picture correspond to the imprint left by the Teflon that was used to generate the initial delamination. Furthermore, the micrographs in Figure 11e–h illustrate darker magnesium bonding surfaces, which are believed to have occurred as a result of the chemical reaction initiated by the elevated exothermic temperature generated as a result of adhesive’s curing process. Furthermore, voids are visible in the adhesive of the specimens that were prepared by the SB bonding method, even with the incorporation of nanoparticles.

Overall, the failure can be classified as the interfacial type. This would suggest that under the present circumstances, one could gain only a marginal enhancement in the interfacial strength as a result of the inclusion of NPs within the interface, unless one could generate a stronger bond between the epoxy adhesive and magnesium substrate, as a result of which the failure mode could be changed into the desirable cohesive failure.

### 3.2. Case Study II

**Figure 12:**
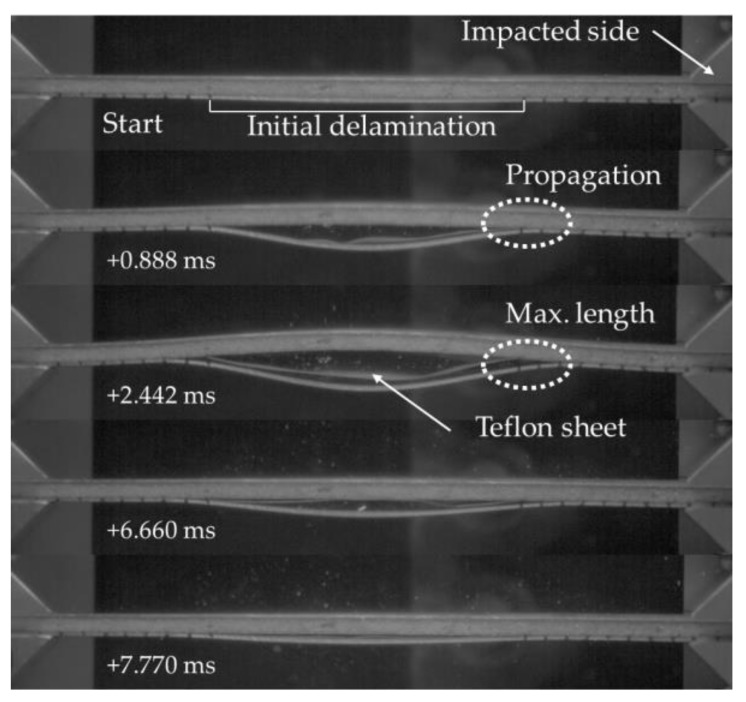
The progressive response of the 3D-FML sandwich under axial impact, for the neat specimens-case study II.

Further insight into the effect of inclusion of GNPs on the mitigation of delamination propagation is gained by reviewing the results of the second case study. The behaviour of the specimens during a typical impact event in shown in Figure 12. Similar to the response of the specimens of the first case study, the specimens remained straight for the first compression phase of the loading, followed by the buckling of the delaminated portion of the skin, which initiated the subsequent delamination propagation stage of the event. Note that the delamination propagated in an unstable manner in specimens that were subjected to an in-plane impact loading. In other words, the delamination remained in its initial state as the specimen experienced the load which increased its curvature up to a certain stage of the event. At that stage, however, the critical stress was reached, causing a sudden incremental elongation of the delamination within the specimens after which the equilibrium was regained, leading to stabilization of the load-end shortening response. Finally, the maximum delamination length was attained, at which stage the entire impact energy was consumed by the specimen, and the impactor bounced back.

**Figure 13:**
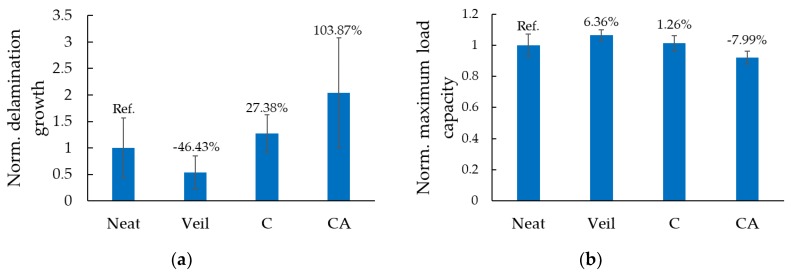
Normalized (**a**) delamination-growth and (**b**) load-bearing capacities for the specimens of case study II (normalized with respect to the “neat” group of specimens).

A comparison of the delamination growth in the tested specimens is illustrated in Figure 13a. The values have been normalized with respect to the average delamination propagation observed in the neat specimens. The delamination is seen to increase with respect to the GNP content, with the worst-case observed when the nanoparticles were added to both the magnesium coating and the resin used to bond the skins to FRP (CA specimens). In those specimens, the final delamination length was twice the length developed in the neat-resin specimens. The best results were achieved when the interface had the fiberglass veil incorporated within. The delamination propagation mitigated in those specimens by an average of 46% when compared to the neat specimens. However, overall, the results exhibit large standard deviations. This is a consequence of the inherently unstable nature of delamination propagation in such brittle mediums. It is worth noting that the standard deviation associated with the specimens that had veiled interface, though relatively large, is the lowest amongst the specimen groups, revealing the slight stabilization of the delamination propagation in those specimens. The observed increase in delamination in specimens containing NPs also corroborates with the observation reported in [52]. Siegfried et al. [52] noted the inclusion of their CNTs led to an increased level of matrix-cracking. This validates our hypothesis that the delamination extends more as the GNP content is increased.

The load-bearing capacity of the specimens of this group is reported in Figure 13b. The incorporation of the fiberglass veil into the interface seems to positively impact the load-bearing capacity by increasing it by 6% and reducing the overall standard deviation of the data. In contrast, when GNPs are included only in the epoxy coating (case C), a marginal improvement of 1% is gained. When the standard deviation values are considered, this 1% enhancement in the capacity cannot be considered as a conclusive measure of improvement. Furthermore, the decrease in load-bearing capacity is more pronounced in the specimens of group CA, corresponding to an 8% reduction.

Overall, one could see that the greatest improvement is observed in the specimens that had a fiberglass veil incorporated between their magnesium skins and FRP core. This procedure led to a significant reduction of delamination propagation by 46% and an increase in load-bearing capacity by 6%. Note that the amount of the required effort in implementing the veil is negligible compared to that consumed by the procedure of dispersing the nanoparticles into the resin, which requires mixing and calendaring efforts. Therefore, it can be appreciated that the suggested veil incorporation technique is the more cost-effective alternative.

**Figure 14:**
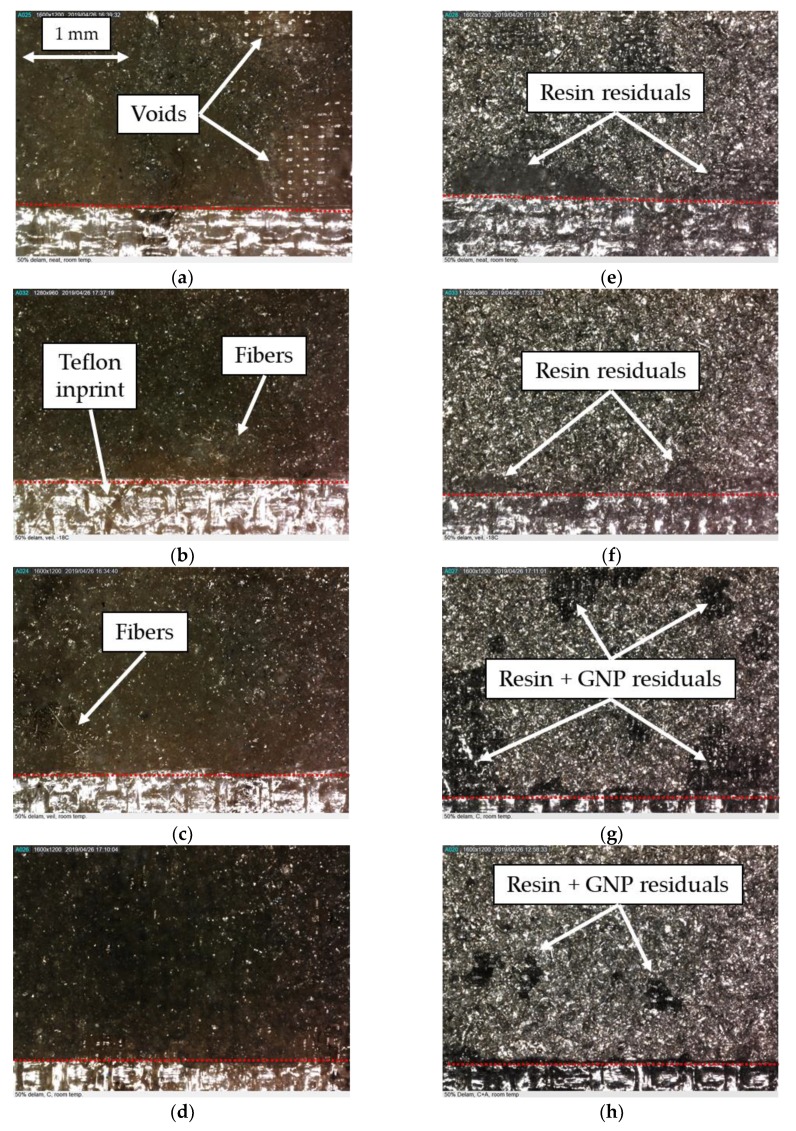
Magnified views of the adherends’ fracture surfaces for specimens of case study II. Images (**a**) to (**d**) show the adhesive interfacial surfaces, while images (**e**) to (**h**) show the magnesium interfacial surfaces. From left to right: specimens “N”, “V”, “C” and “CA”.

Similarly to what was done for case study I, micrographs of the bonding surfaces for case study II are provided in Figure 14. Compared to the previous study case, fewer voids are visible at the interface of the specimens that were prepared by the SBC bonding method. In fact, in the latter case, the voids seem to exist only in the specimens that were assembled with the neat adhesive. These observations indicate that the use of nanoparticles and the veil resulted in a more homogeneous distribution of the resin during the curing process.

Moreover, the relatively small regions of resin residuals visible on the magnesium interface surfaces (see Figure 14e) suggest that the delamination initiated and propagated mainly at the interface between the magnesium skins and the resin layer. 

Please note the relatively darker colour regions of resin residuals that can be seen in two cases (i.e., Figure 14g,h). The two cases correspond to the specimens that were prepared by the SBC bonding method and containing GNPs (i.e., specimens C and CA). The darker colour is believed to represent regions with a higher concentration (agglomeration) of nanoparticles developed in specimens that contained GNP only in the coating and in both coating and adhesive, respectively.

In this case, the darker magnesium bonding surfaces reported for case study I are mitigated using the new bonding method and incorporation of the cold cure adhesive, which is believed to have improved the interface compatibility, thus increasing the interface strength of the specimens of this case study. However, even with the new surface preparation method, the failure mode remains as an interfacial type. In comparison, more consistent and relatively substantive improvements could be gained by the inclusion of the more cost-effective fiberglass veil in the interface.

### 3.3. Case Study III

The last case study aims to investigate the effect of sub-freezing temperature on the performances of the 3D-FML hosting a delamination and whether the interfacial delamination resistance could be enhanced by the inclusion of GNPs. For this, the specimens of this case study were tested at a quasi-static loading rate of 0.5 mm/min. The imposed displacement of 1.4 mm facilitated the desired state of delamination propagation without causing the complete failure of the specimens (similar to what was done in the second case study). As stated earlier, the sub-freezing environment of this case study was generated by using liquid nitrogen, hence, the specimens of this case study are referred to as the LN2 specimens, and the specimens tested at room temperature are referred to as RT specimens.

**Figure 15:**
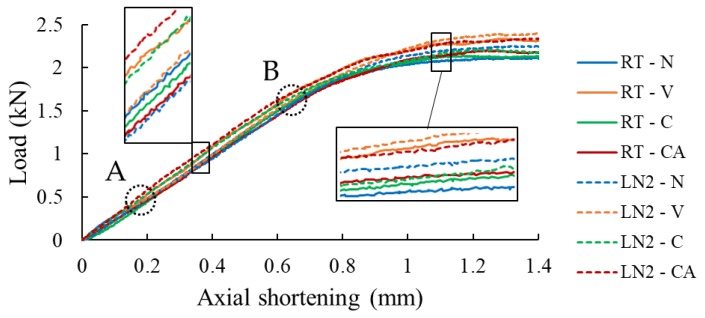
Axial load vs. axial shortening curves. Point A corresponds to the onset of buckling of the delaminated skin, while point B corresponds to the onset of the global buckling of the specimen.

**Figure 16:**
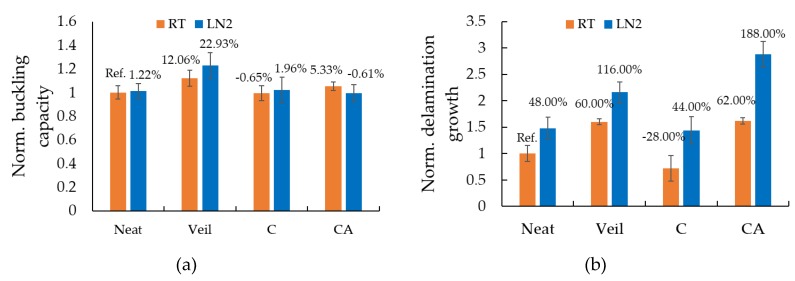
(**a**) normalized buckling capacity and (**b**) normalized delamination growth length for specimens tested at room and −50 °C.

The qualitative response of the specimens was identical to the behaviour described for the specimens of case study II as was illustrated in Figure 12; therefore, for the sake of space, the images are not presented. However, the quantitative results of the static buckling tests conducted both at room and sub-freezing temperatures are reported in Figure 15 and Figure 16. The results illustrated in Figure 15 reveal that the LN2 specimens show vary similar stiffness compared with the RT specimens. However, the load-bearing capacity seems to be slightly higher for the LN2 specimens. Moreover, no clear distinction between the responses of neat and GNP-reinforced specimens can be seen, except for the case of LN2-CA specimens, which show slightly higher stiffness compared to the other LN2 specimens. Moreover, similar to the performance of specimens of case study II, the specimens hosting the fiberglass veil exhibited the best performances amongst the tested specimens in terms of buckling capacity at both room and sub-freezing temperatures. 

To facilitate an easier comparison, the normalized buckling load capacities are reported in Figure 16a. The values are normalized with respect to the average value corresponding to the neat specimens tested at room-temperature (RT-N). The buckling load was taken as the load at which the linear slope of the load-displacement curve changes to a non-linear one (see point B on the graphs of Figure 15). The results also reveal that the inclusion of nanoparticles had a negligible effect on the buckling capacity of the specimens tested at both temperatures, reaching a maximum of approximately 5% for the RT-CA specimens. On the contrary, the more cost-effective inclusion of the veil within the interface increased the buckling capacity by 12% and 22%, respectively for specimens tested at RT and −50 °C, respectively.

In addition, the normalized delamination propagation response of the specimens are reported in Figure 16b (results normalized with respect to the RT-N case). The sub-freezing temperature caused the delamination to grow to a greater length compared to the response observed at RT. Specifically, the delamination length increased by 48%, 35%, 100%, and 78% for the specimens of categories neat, veil, C, and CA, respectively. Interestingly, while the presence of the interface veil reduced the growth of delamination by 28% when specimens were tested at RT, the veil’s effect diminished significantly in specimens that were tested at the sub-freezing temperature; nonetheless, the veil still helped to suppress the delamination growth when compared to the growth observed in specimens that did not have the veil at their interfaces. It can also be seen that although the test results (i.e., load-axial shortening curves) are very consistent and have very low standard deviations, nevertheless, the standard deviations are relatively large when considering the delamination length results. This observation further validates our earlier statement that such large standard deviations are inherent to delamination growth being an unstable phenomenon in brittle materials. Also, similar to the results seen in the other case studies, the use of the veil resulted in the highest overall buckling capacity and the highest delamination mitigation, with the proviso that its effectiveness becomes adversely impacted by the sub-freezing temperature.

Finally, please note that case study III’s bonding surface micrographs are omitted because they were very similar to those shown in Figure 14, thus not further information would be provided.

## 4. Summary and Conclusions

A systematic investigation was conducted to examine the effect of graphene nanoplatelets (GNPs) used to reinforce a structural epoxy resin. The resin was used to mate the magnesium skins and composite core of a recently developed 3D fiber-metal laminate (3D-FML). The response of the resin and interface strength in the 3D-FML specimens were evaluated by subjecting the specimens to compressive loading at quasi-static and impact loading rates. Therefore, the impact buckling strength, delamination buckling strength, and delamination propagation were used as the evaluation criteria in this study. Two different techniques were used to join the skins to the FRP core. In the first method, the skins were directly bonded to the core using a hot-cure structural resin, with the mating skins’ surfaces prepared by the conventional abrasive (sandblasting) method. In the second method, a cold-cure less expensive structural resin was used, and a newly developed resin coating method was employed for preparing the skins’ mating surface. The specimens prepared using the first technique (i.e., case study I specimens), were axially impacted at four energies (1.5 J. 3 J, 4.5 J and 7 J). Two different case studies were organized to examine the effect of initial delamination present in such 3D-FMLs by considering intact specimens and specimens with a delamination length of 30%, 50% and 70% (percentiles refer to the ratio of delamination length to specimen’s gage length). Moreover, GNP contents of 0.5 wt%, 1 wt% and 2 wt% were used to reinforce the resin in this study. The results from the first case study can be summarized as follows:

- The presence of initial delamination greatly affected the load-bearing capacity of the specimens, but its length had a negligible effect.
- For the intact specimens (i.e., with no initial delamination), the incorporation of GNPs showed its maximum enhancing effect when the specimens were subjected to the highest impact energy (7 J). The observed enhancements were 12.5%, 10.9%, and 10.7% corresponding to GNP contents of 0.5 wt%, 1 wt%, and 2 wt%, respectively. Ironically, a degradation of the strength was noted in specimens that were subjected to 4.5 J impact energy.
- Among the specimens that hosted a delamination, the specimens that were reinforced with 0.5 wt% of GNP content exhibited the most gain in strength under three out of the four impact energies tried. The exceptions were the specimens that were subjected to 4.5 J impact energy, for which 2 wt% GNP content produced the best results.
- Microscopic examination revealed the existence of some voids at the bonding interface of the 3D-FMLs.

To further explore the effect of GNP inclusion on the performance of the magnesium/FRP interface (and overall 3D-FML), additional case studies were considered. In the second case study, the specimens had the optimum GNP content of 0.5 wt%, with a fixed initial delamination of 50%, all tested under 2.85 J impact energy. The outcome of this case study is summarized as follows:

- The delamination propagated in an unstable manner.
- A higher GNP content led to a higher delamination length, with a 100% increase in delamination growth observed in the CA specimens.
- The use of a fiberglass veil interleaved between the magnesium and the FRP core mitigated the delamination extension by an average of 46% and increased the load-bearing capacity by 6%.
- The GNPs inclusion produced either no effect on the load capacity of most specimens or led to even negative effect in some (a reduction of 8% was observed in the CA specimens).
- The void content in the bonding region was drastically reduced when the SBC method was employed and voids were completely nullified when the veil or GNPs were incorporated within the interface; nonetheless, the delamination growth persisted owing to the lack of optimal chemical compatibility between magnesium and epoxy resin.

Finally, an investigation was carried out in a case study (III) examining the effect of sub-freezing temperature (−50 °C) on the delamination buckling and propagation of the 3D-FML and the effect of GNP inclusion. The specimens within this case study were tested under a quasi-static loading rate. The results are summarized as follows:

- The specimens’ apparent stiffness changed marginally when exposed to the sub-freezing temperature.
- The buckling load capacity was positively affected by the sub-freezing temperature, especially when the veil was used.
- The sub-freezing environment caused an increase in delamination growth, especially in the GNP-reinforced specimens.

Overall, it can be concluded that some improvement in performances could be gained by incorporating GNPs in the interface of the 3D-FMLs; however, one could also expect degradation of the performance under certain circumstances. In comparison, incorporation of the fiberglass veil as demonstrated in this study would be a more effective and less costly means for enhancing the performance of 3D-FMLs under in-plane compressive loading. Not only is the cost of the veil lower than that of GNPs, but the labor cost associated with its incorporation would be much less than that required for processing GNPs into the resin. 

In closing, the lack of the expected gain in performance as a result of reinforcing the resin with GNP is believed to be due to the lack of chemical compatibility between the resin and magnesium. The incompatibility does not allow the GNPs to demonstrate their full potential in enhancing the strength of the interface resin. This is mainly because the failure along the interface is in the interfacial mode (failure or resin/magnesium interface), as opposed to being of a cohesive type. Therefore, it is strongly believed that future works should focus on improving the chemical compatibility between the resin and magnesium. Based on the results of this study and those reported in the literature, it is strongly believed that once the interface compatibility issue is resolved, the incorporation of nanoparticles will positively and significantly influence the interface strength and hence the overall performance of 3D-FMLs when subject to in-plane loadings.

## Abbreviations and Acronyms

3D-FGF	3D fiberglass fabric
3D-FML	3D fiber-metal laminate
C	nanoparticles included in the coating
CA	nanoparticles included in the coating and the adhesive
CNT	carbon nano-tubes
FML	fiber-metal laminate
FRP	fiber-reinforced polymer
GNP	graphene nanoplatelets
LN2	liquid nitrogen
N	specimens with neat resin
ND	no initial delamination
NP	nanoparticles
RMS	root-mean square
RT	room temperature
V	fiberglass veil
wt%	weight percentage
x%	percentage of initial delamination
Note	“s” following above acronyms make them plural
